# Detecting the invisible: catatonia in autoimmune encephalitis and how to intervene early

**DOI:** 10.1192/j.eurpsy.2026.10314

**Published:** 2026-07-31

**Authors:** F. J. Cruz Aviña

**Affiliations:** Liason psychiatry, Centro Medico Nacional “20 de Noviembre”, Mexico City, Mexico

## Abstract

**Abstract:**

Anti-LGI1 autoimmune encephalitis can initially present with affective, psychotic, or behavioral symptoms, leadingto misdiagnosis as a primary psychiatric condition. Catatonia associated with this entity is rare and oftenunderrecognized. We report a case of delayed diagnosis, where catatonia emerged in the context of a progressiveneuropsychiatric syndrome later confirmed as anti-LGI1 autoimmune encephalitis

A 49-year-old male presented with an 8-month evolution of neuropsychiatric symptoms, including panic attacks,hallucinations, disorganized behavior, and faciobrachial dystonic seizures. He received multiple psychiatrictreatments with no sustained improvement. In early 2025, neurology evaluation included a brain MRI showingcortical atrophy and temporal FLAIR hyperintensities; EEG showed diffuse delta slowing and temporal sharp waves.CSF analysis revealed elevated protein levels without pleocytosis. Serology confirmed anti-LGI1 antibodies. InMarch 2025, psychiatry assessed catatonia using the Bush-Francis Catatonia Rating Scale (BFCRS), scoring 10 onscreening and 18 on severity. Treatment with lorazepam and quetiapine, and later instalation of memantine led to significant clinical improvement.Rituximab was later administered with favorable neurological stabilization.

The patient exhibited catatonic features including fixed gaze, rigidity, echolalia, automatic obedience, stereotypies,and disorganized speech. Marked clinical improvement followed immunotherapy and benzodiazepines.

This case highlights the importance of considering autoimmune encephalitis in patients with atypical psychiatricsymptoms and poor response to psychotropics. Early neurological evaluation and systematic screening forcatatonia can significantly improve diagnostic accuracy and outcomes. Multidisciplinary collaboration is crucial foridentifying catatonia as a possible manifestation of underlying neuroinflammatory disorders.

**Disclosure of Interest:**

None Declared

